# Severe hypereosinophilia and eosinophilic myocarditis during secondary plasma cell leukemia: evidence of a paraneoplastic manifestation of aggressive myeloma evolution

**DOI:** 10.1007/s00277-026-07172-0

**Published:** 2026-07-09

**Authors:** Maria Elisabetta Catini, Matteo Garibotto, Margherita Castiglione Morelli, Alberto Tagliafico, Soncini Debora, Fabio Guolo, Roberto Massimo Lemoli, Antonia Cagnetta, Michele Cea

**Affiliations:** 1 Clinic of Hematology, Department of Internal Medicine and Medical Specialties (DiMI), University of Genoa, Genoa, Italy; 2 Clinic of Hematology, IRCCS Azienda Ospedaliera Metropolitana, Ospedale Policlinico San Martino, Genoa, Italy; 3 Department of Radiology and Interventional Radiology, IRCCS AOU San Martino, Genoa, Italy; 4 Department of Internal Medicine and Specialities (DiMI), University of Genoa, Viale Benedetto XV n.6, Genoa, 16132 Italy

**Keywords:** Multiple myeloma, Secondary plasma cell leukemia, Hypereosinophilia, Eosinophilic myocarditis, Loeffler syndrome, Cardiac magnetic resonance

## Abstract

Secondary plasma cell leukemia (sPCL) represents one of the most aggressive forms of Multiple Myeloma (MM) progression and remains associated with poor outcomes despite the availability of novel immunotherapeutic approaches. Severe hypereosinophilia is exceptionally rare in plasma cell neoplasms and may occasionally occur as a paraneoplastic manifestation of aggressive disease biology. In selected cases, eosinophilic infiltration can result in life-threatening organ damage, particularly involving the cardiovascular system. We report the case of a 40-year-old woman with IgG lambda MM who achieved complete remission following daratumumab-based induction therapy, autologous stem cell transplantation, and lenalidomide maintenance. Approximately one year after transplantation, she developed rapidly progressive relapse characterized by constitutional symptoms, circulating clonal plasma cells, extensive bone marrow involvement, severe hypereosinophilia, and marked cardiac biomarker elevation. Comprehensive infectious, autoimmune, and molecular investigations excluded alternative causes of eosinophilia. Cardiac magnetic resonance imaging revealed diffuse myocardial edema, increased myocardial wall thickness, extensive subendocardial late gadolinium enhancement, regional wall motion abnormalities, and left ventricular mural thrombosis, findings highly suggestive of eosinophilic myocarditis with endomyocardial involvement consistent with Loeffler syndrome. Urgent cytoreductive therapy with cyclophosphamide and dexamethasone resulted in rapid normalization of eosinophil counts, substantial clinical improvement, and reduction of cardiac symptoms, strongly supporting a pathogenetic relationship between eosinophilia and plasma cell disease burden. Despite subsequent salvage therapies, including isatuximab-based treatment and the BCMA-directed bispecific antibody elranatamab, disease control remained transient and the patient ultimately died from refractory sPCL. This case highlights severe hypereosinophilia as a rare paraneoplastic manifestation of aggressive clonal evolution in MM and emphasizes the importance of prompt recognition of eosinophilic cardiac involvement. The close correlation between eosinophil kinetics and disease activity further suggests that hypereosinophilia may represent a potential clinical indicator of aggressive plasma cell disease evolution and warrants further investigation.

## Introduction

Multiple myeloma (MM) is a biologically heterogeneous plasma cell malignancy characterized by clonal plasma cell expansion within the bone marrow and progressive end-organ damage [1–3]. Over the past two decades, the introduction of proteasome inhibitors, immunomodulatory agents, monoclonal antibodies, cellular therapies, and T-cell-engaging strategies has substantially improved patient outcomes [4]. Nevertheless, a subset of patients develops highly aggressive disease characterized by treatment resistance, rapid relapse, extramedullary dissemination, or leukemic transformation. Secondary plasma cell leukemia (sPCL) represents one of the most aggressive evolutionary phases of MM and is associated with high tumor burden, profound genomic instability, treatment refractoriness, and poor survival [5]. Recent revisions of diagnostic criteria have expanded the recognition of circulating plasma cells as a marker of biologically aggressive disease, underscoring the continuum between advanced MM and leukemic transformation [6,7]. Despite therapeutic advances, outcomes remain dismal once sPCL develops [8]. 

Hypereosinophilia, conventionally defined as an absolute eosinophil count ≥ 1.5 × 10^9/L, is most commonly associated with allergic disorders, parasitic infections, drug-induced reactions, autoimmune diseases, and clonal eosinophilic neoplasms. Persistent eosinophilia is clinically relevant because eosinophilic infiltration may result in progressive tissue injury involving the skin, lungs, gastrointestinal tract, nervous system, and cardiovascular system. Among these manifestations, cardiac involvement represents the most severe complication and remains a major cause of morbidity and mortality in hypereosinophilic syndromes. Eosinophilic cardiac disease encompasses a spectrum ranging from acute eosinophilic myocarditis to the classical eosinophilic endomyocarditis described by Loeffler. The pathological process is characterized by eosinophil-mediated myocardial injury, followed by mural thrombosis and, ultimately, endomyocardial fibrosis leading to restrictive cardiomyopathy. Cardiac magnetic resonance imaging (CMR) has emerged as a key non-invasive diagnostic modality, allowing detailed characterization of myocardial inflammation, edema, fibrosis, and intracardiac thrombotic complications [9, 10].

The coexistence of marked hypereosinophilia and plasma cell neoplasms is exceedingly uncommon. Only sporadic cases of MM associated with eosinophilia have been reported, and the underlying biological mechanisms remain poorly understood [11–14]. Proposed explanations include cytokine-mediated eosinophilopoiesis driven by interleukin-5 (IL-5), interleukin-3 (IL-3), granulocyte-macrophage colony-stimulating factor (GM-CSF), and other inflammatory mediators produced either by malignant plasma cells or by the dysregulated bone marrow microenvironment [15–17]. Emerging evidence additionally suggests complex bidirectional interactions between eosinophils and plasma cells within the marrow niche, raising the possibility that eosinophilia may represent more than a simple reactive phenomenon [18, 19].

Here, we describe a rare case of MM evolving into secondary plasma cell leukemia accompanied by severe paraneoplastic hypereosinophilia and eosinophilic myocarditis with endomyocardial thrombosis. The close temporal relationship between eosinophil counts and disease activity, together with the rapid resolution of eosinophilia following anti-myeloma therapy, strongly supports a pathogenetic link between aggressive plasma cell disease and eosinophilic activation. Furthermore, this case highlights the pivotal role of advanced cardiac imaging in identifying potentially life-threatening eosinophilic cardiac involvement in patients with rapidly progressive hematologic malignancies.

## Case presentation

**Fig. 1:**
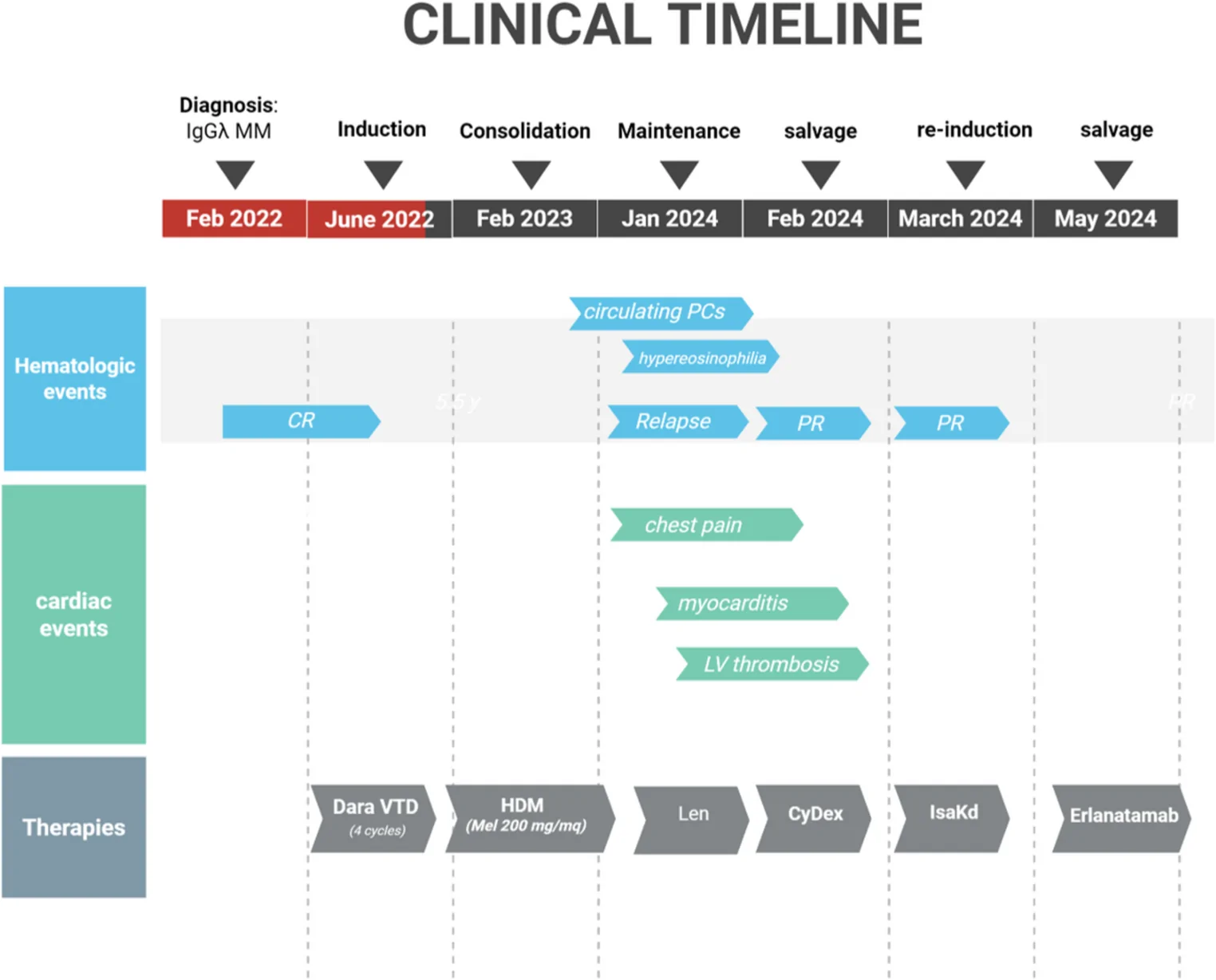
Clinical timeline of disease evolution. Timeline summarizing the clinical course from the initial diagnosis of IgG lambda MM in February 2022 through autologous stem cell transplantation, maintenance therapy, relapse, development of sPCL, severe hypereosinophilia, eosinophilic myocarditis, subsequent salvage therapies, and eventual disease progression. Severe hypereosinophilia and eosinophilic myocarditis are shown as events of the relapse phase (January 2024); no eosinophilia was documented during the preceding induction, consolidation, or maintenance periods, consistent with the eosinophil kinetics depicted in Fig. 2

**Fig. 2:**
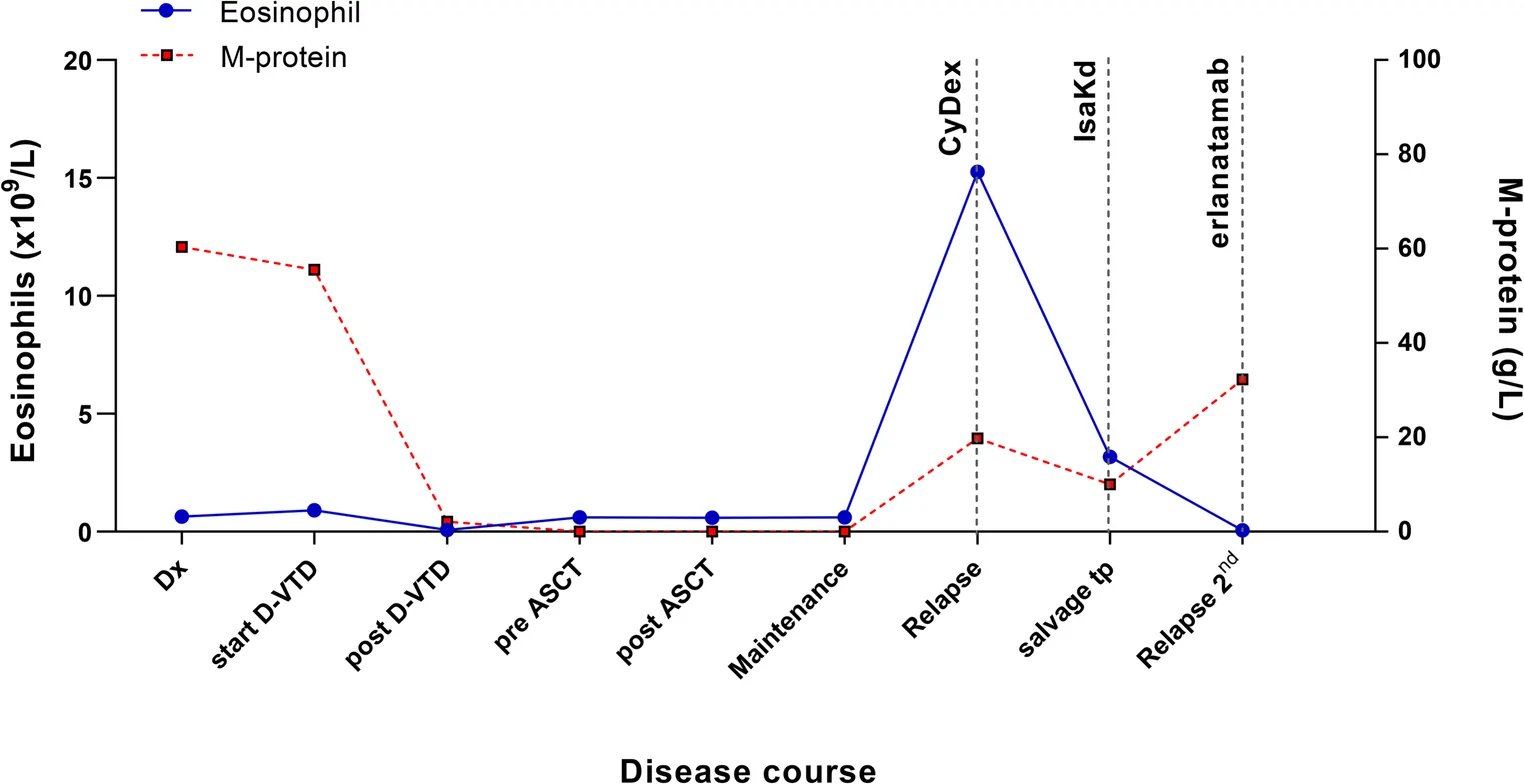
Relationship between eosinophil count and plasma cell disease activity. Temporal evolution of absolute eosinophil count, monoclonal protein levels, and major therapeutic interventions during disease progression. Severe hypereosinophilia developed concomitantly with leukemic transformation and rapidly normalized following cyclophosphamide-dexamethasone cytoreduction, suggesting a close relationship between eosinophilia and plasma cell disease burden

A 40-year-old woman was diagnosed with IgG lambda MM in February 2022 following evaluation for symptomatic plasma cell disease. (Fig. 1) At diagnosis, cytogenetic and fluorescence in situ hybridization (FISH) analyses did not reveal high-risk abnormalities. Given her young age and transplant eligibility, she received induction therapy with daratumumab, thalidomide, bortezomib, and dexamethasone (D-VTD), achieving complete biochemical remission and marked clinical improvement. In February 2023, she underwent autologous stem cell transplantation (ASCT) following high-dose melphalan conditioning (HD-MEL200). Post-transplant evaluation confirmed sustained response, and maintenance treatment with lenalidomide was initiated. The patient remained clinically stable until January 2024, when she developed rapidly progressive fatigue, intermittent fever, exertional dyspnea, diffuse arthralgia, peripheral edema, and new-onset chest pain. These symptoms were accompanied by biochemical evidence of aggressive disease relapse. Serum monoclonal protein increased from 4.1 g/L to 19.8 g/L, together with worsening lambda free light-chain predominance. Complete blood count demonstrated leukocytosis (15.98 × 10^9/L), anemia (hemoglobin 8.0 g/dL), and thrombocytopenia (44 × 10^9/L). A particularly striking finding was the presence of severe hypereosinophilia. The absolute eosinophil count was 5.26 × 10^9/L at presentation and rapidly increased to a peak value of 16.7 × 10^9/L over the following days. (Fig. 2)

Peripheral blood smear revealed numerous mature eosinophils with preserved granulation and bilobed nuclei, together with circulating atypical plasma cells. Of note, hypereosinophilia was not present during the earlier post-transplant and maintenance phases and emerged only at the time of relapse, rising in parallel with the circulating clonal plasma cell burden (Fig. 2). Flow cytometric immunophenotyping identified approximately 15% circulating clonal plasma cells expressing CD138++, CD38+, CD56+, and lambda light-chain restriction, in the absence of CD19 and CD20 expression. Bone marrow examination demonstrated extensive plasma cell infiltration involving approximately 50% of marrow cellularity. In the setting of circulating clonal plasma cells, extensive bone marrow involvement, rapidly increasing monoclonal protein levels, worsening cytopenias, and clinical deterioration, the overall picture was considered consistent with leukemic transformation of MM and sPCL according to contemporary diagnostic criteria [7]. 

Given the severity of eosinophilia, an extensive diagnostic workup was undertaken to exclude alternative causes. Serological and parasitological investigations for *Strongyloides stercoralis*, *Toxocara* spp., *Schistosoma* spp., and *Trichinella* spp. were negative. Viral studies for HIV, hepatitis B virus, cytomegalovirus, Epstein–Barr virus, HHV-7, and HHV-8 were also negative [20–24]. Autoimmune screening, including antinuclear antibodies, anti-double-stranded DNA antibodies, and rheumatoid factor, showed no abnormalities. Serum tryptase and vitamin B12 levels were within normal limits. Molecular analyses excluded BCR/ABL1, FIP1L1/PDGFRA rearrangements, and JAK2 mutations. No recent drug exposure, allergic condition, or epidemiological risk factor for parasitic infection was identified. Lenalidomide-associated eosinophilic toxicity was considered in the differential diagnosis [25–28]. However, the patient had tolerated maintenance treatment for several months without eosinophilic manifestations, and eosinophilia developed concurrently with abrupt hematologic progression. Moreover, eosinophil counts closely paralleled disease activity and rapidly normalized following anti-myeloma therapy. No clinical or radiological findings suggestive of eosinophilic drug hypersensitivity syndrome or eosinophilic pneumonia were observed. Collectively, these findings strongly supported a diagnosis of reactive paraneoplastic hypereosinophilia associated with plasma cell disease progression.

**Fig. 3:**
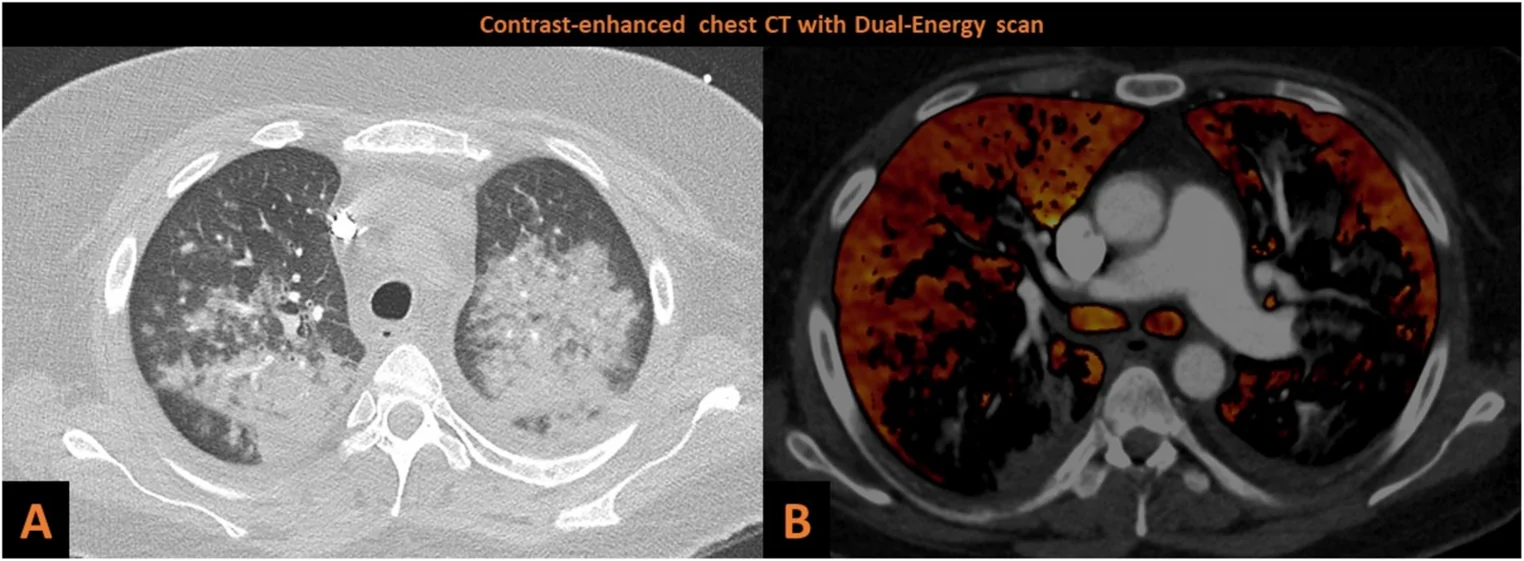
Dual-energy contrast-enhanced thoracic computed tomography. **A** Axial chest CT image showing extensive bilateral pulmonary consolidations associated with bilateral pleural effusions. **B** Corresponding iodine distribution map obtained with dual-energy acquisition demonstrating the absence of perfusion defects suggestive of pulmonary embolism. Imaging findings were interpreted as inflammatory pulmonary involvement rather than thromboembolic disease

**Fig. 4:**
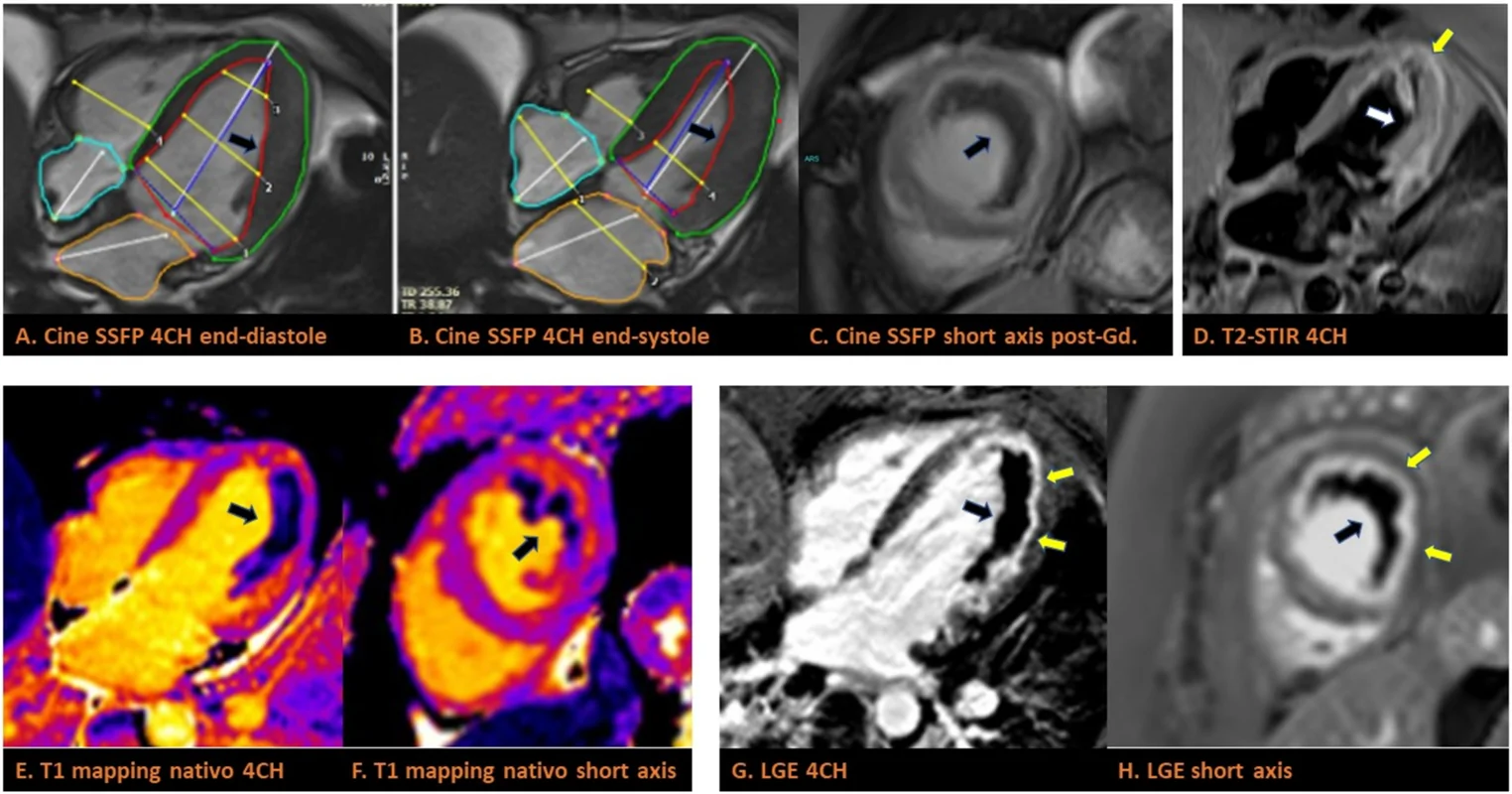
Cardiac magnetic resonance imaging findings consistent with eosinophilic myocarditis and Loeffler endocarditis. **A**, **B** Cine steady-state free precession (SSFP) sequences demonstrating increased myocardial wall thickness involving the mid-ventricular and apical anterolateral segments (black arrows). **C** Post-contrast cine SSFP image showing regional hypokinesia of the distal left ventricular free wall associated with extensive mural thrombotic apposition along the endocardial surface (black arrow). **D** T2-weighted STIR sequence demonstrating diffuse myocardial edema involving the apical segments (yellow arrow) and the interface between the ventricular cavity and the anterolateral wall (white arrow). **E**, **F** Native myocardial T1 mapping images showing abnormal tissue characteristics in the corresponding myocardial regions (black arrows). **G**, **H** Late gadolinium enhancement sequences revealing extensive subendocardial enhancement involving the mid-ventricular and apical left ventricular wall and apex (yellow arrows), together with large endocardial thrombotic deposits (black arrows). The combination of myocardial edema, diffuse subendocardial injury, and mural thrombosis is highly suggestive of eosinophilic myocarditis with endomyocardial involvement

Because of persistent chest pain, progressive dyspnea, peripheral edema, and marked elevation of cardiac biomarkers, urgent cardiologic evaluation was performed. Troponin levels were markedly elevated (9067 ng/L), while brain natriuretic peptide (BNP) reached 14,500 pg/mL. Transthoracic echocardiography demonstrated mildly impaired left ventricular systolic function with an estimated ejection fraction of approximately 50%. Prior to cardiac magnetic resonance imaging, contrast-enhanced dual-energy thoracic computed tomography was performed because of worsening dyspnea and chest pain, revealing bilateral pulmonary consolidations and pleural effusions without evidence of pulmonary embolism (Fig. 3). To further characterize the myocardial injury, cardiac magnetic resonance imaging was performed. Cine steady-state free precession (SSFP) sequences demonstrated increased myocardial wall thickness involving the mid-ventricular and apical anterolateral segments, associated with regional hypokinesia of the distal left ventricular free wall. Post-contrast cine images revealed extensive mural thrombotic apposition along the left ventricular endocardial surface. T2-weighted short tau inversion recovery (STIR) sequences showed diffuse myocardial hyperintensity involving the apical segments and the interface between the ventricular cavity and the anterolateral wall, consistent with significant myocardial edema and active inflammation. Native T1 mapping demonstrated abnormal myocardial tissue characteristics in the corresponding regions. Late gadolinium enhancement (LGE) sequences revealed diffuse subendocardial enhancement predominantly involving the mid-ventricular and apical left ventricular wall as well as the apex, associated with extensive endocardial thrombotic deposits. (Fig. 4) Taken together, the coexistence of severe hypereosinophilia, markedly elevated cardiac biomarkers, diffuse myocardial edema, characteristic subendocardial enhancement, regional contractile abnormalities, and intracardiac thrombosis was considered highly suggestive of eosinophilic myocarditis with endomyocardial involvement, consistent with Loeffler syndrome. Although endomyocardial biopsy remains the diagnostic gold standard, invasive sampling was not pursued because the combination of clinical, laboratory, and imaging findings provided a high degree of diagnostic certainty and was unlikely to alter immediate therapeutic management.

**Fig. 5:**
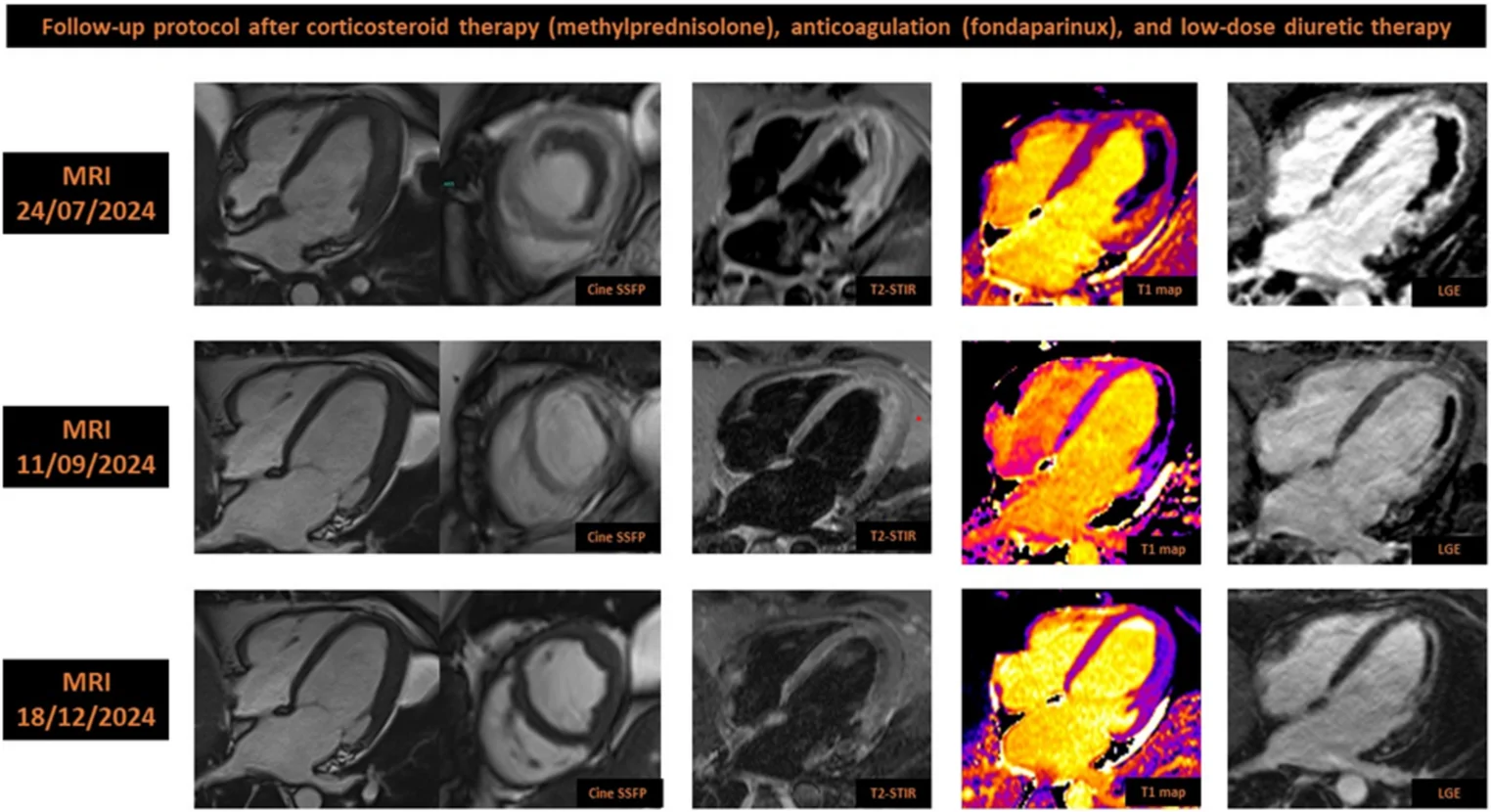
Follow-up cardiac magnetic resonance imaging after treatment. Serial CMR examinations performed in September and December 2024 following corticosteroid therapy, anticoagulation, and anti-myeloma treatment. Images demonstrate the evolution of myocardial abnormalities and thrombotic burden over time, allowing assessment of treatment response and disease stabilization

Given the simultaneous presence of aggressive hematologic relapse and evolving cardiac injury, urgent cytoreductive therapy with cyclophosphamide and dexamethasone was initiated. A rapid clinical and hematologic response followed. Within ten days, eosinophil counts normalized completely, chest pain resolved, dyspnea substantially improved, and overall performance status recovered. The close temporal association between anti-myeloma treatment and eosinophil normalization strongly supported a direct relationship between eosinophilia and plasma cell disease burden. Anticoagulation with low-molecular-weight heparin was initiated because of intracardiac thrombosis. Follow-up cardiac magnetic resonance imaging performed in September and December 2024 demonstrated stabilization and partial regression of the previously documented inflammatory and thrombotic abnormalities, consistent with treatment response and resolution of active eosinophilic myocardial injury (Fig. 5). After initial stabilization, salvage treatment with isatuximab, carfilzomib, and dexamethasone was started. However, carfilzomib was subsequently discontinued because of concerns regarding potential cardiotoxicity in the context of recent myocarditis and borderline ventricular function. Following further disease progression, treatment with the BCMA-directed bispecific antibody elranatamab was initiated. Although a transient clinical response was observed, disease control proved short-lived. The patient ultimately developed rapidly progressive refractory secondary plasma cell leukemia and died from disease progression.

## Discussion

This case illustrates a rare association between secondary plasma cell leukemia (sPCL), severe paraneoplastic hypereosinophilia, and eosinophilic myocarditis with endomyocardial thrombosis. Four aspects deserve attention: the rarity of eosinophilia in plasma cell neoplasms, its close temporal relationship with disease burden, the extensive cardiac involvement documented by CMR, and the biological implications of eosinophilia as a marker of aggressive disease evolution.

Hypereosinophilia is uncommon in MM, where eosinophilia more often reflects allergic, parasitic, drug-related, autoimmune, or clonal eosinophilic disorders, so its appearance warrants a comprehensive evaluation. In our patient, extensive infectious, autoimmune, and molecular investigations excluded an alternative cause, and eosinophilia coincided with rapid progression marked by rising monoclonal protein, extensive marrow infiltration, circulating plasma cells, and clinical deterioration, supporting a paraneoplastic origin. Lenalidomide-associated toxicity, the principal alternative, was considered unlikely for the reasons detailed above.

The biological link between eosinophilia and plasma cell neoplasms remains incompletely understood. Malignant plasma cells may stimulate eosinophilopoiesis through cytokines such as IL-5, IL-3, and GM-CSF, or advanced MM may remodel the marrow microenvironment, producing aberrant inflammatory signaling and eosinophil expansion. Reciprocal eosinophil–plasma cell interactions within the marrow niche have also been described, whereby eosinophils provide survival signals to plasma cells while malignant plasma cells promote eosinophil recruitment, suggesting eosinophilia may reflect a dysregulated tumor microenvironment rather than a purely reactive epiphenomenon. The kinetics observed here support this view: hypereosinophilia appeared with leukemic transformation, peaked at maximal disease burden, and normalized rapidly after cytoreduction. Although not generalizable from a single case, this raises the hypothesis that eosinophilia may accompany aggressive clonal evolution in a subset of plasma cell neoplasms and merits study in larger cohorts. Only a few cases of plasma cell neoplasms with marked eosinophilia have been reported, and concomitant eosinophilic cardiac involvement is exceptionally rare (Table 1). To our knowledge, this is among the very few reported cases of sPCL with severe hypereosinophilia and the first with detailed CMR characterization of eosinophilic myocarditis in this setting.

**Table 1:** Reported cases of plasma cell neoplasms presenting with hypereosinophilia

Author	Year	Plasma cell disorder	Peak eosinophil count (×10⁹/L)	Organ involvement	Outcome
Franchi F. et al. [11]	1984	Plasmacytoma	N/A	No	Alive
Glantz L. et al. [12]	1995	MM	109.7 × 10⁹/L	No	Poor
Robier C. et al. [13]	2015	sPCL	8.85 × 10^9^/L	No	Poor
Zhong J. et al. [14]	2026	MM	N/A	No	Alive
Catini E. et al.	2026	sPCL	16.7 × 10^9/L	Eosinophilic myocarditis	Death

Abbreviations: *MM*, multiple myeloma; *sPCL*, secondary plasma cell leukemia; *N/A*, not available

The extent of cardiac involvement is a particularly relevant aspect, being the most severe complication of hypereosinophilic syndromes and a major prognostic determinant. Eosinophilic myocardial injury classically progresses through three overlapping stages: an initial necrotic phase driven by eosinophil granule proteins, a thrombotic phase with mural thrombosis, and a fibrotic phase leading to restrictive cardiomyopathy. Here, the diffuse subendocardial enhancement was characteristic of eosinophilic endomyocardial disease and distinct from the vascular distribution of ischemic cardiomyopathy, while coexisting myocardial edema and intracardiac thrombosis suggested imaging during the necrotic-to-thrombotic transition of Loeffler endocarditis, characterization difficult to obtain with echocardiography alone. Although endomyocardial biopsy remains the histopathological gold standard, its yield is limited by sampling error and it carries risk in unstable patients; the combination of marked hypereosinophilia, elevated cardiac biomarkers, characteristic CMR findings, and exclusion of alternative causes is widely considered sufficient to support a highly probable diagnosis when biopsy is not feasible, and the rapid improvement after cytoreduction further reinforced this interpretation.

The course also highlights the aggressiveness of sPCL. Despite prompt cytoreduction, anti-CD38-based salvage therapy, and subsequent BCMA-directed immunotherapy, disease control remained transient, consistent with the persistently poor survival reported in sPCL despite novel agents. Profound hypereosinophilia in this context may thus represent an additional feature of highly aggressive disease biology.

Several clinical implications follow. Unexplained eosinophilia in MM should not be reflexively attributed to treatment toxicity or reactive causes; its emergence may warrant urgent reassessment for aggressive progression, including leukemic transformation; cardiac involvement should be actively investigated when eosinophilia is accompanied by chest pain, dyspnea, biomarker elevation, or heart failure, since early recognition may prevent irreversible myocardial damage; and the rapid normalization of eosinophils after anti-myeloma therapy underscores the importance of prompt cytoreduction in eosinophil-mediated organ injury. This report has limitations. Endomyocardial biopsy was not performed, so histologic confirmation was not obtained and the diagnosis remains highly probable rather than definitive, although supported by marked hypereosinophilia, elevated cardiac biomarkers, characteristic CMR findings, exclusion of alternative causes, and rapid improvement after cytoreduction. Cytokine profiling (e.g., IL-5, IL-3, GM-CSF) was not performed, so the underlying mechanisms remain speculative, and molecular/genomic characterization of the leukemic transformation was limited. Further studies are needed to clarify this association and to determine whether eosinophilia has prognostic or disease-monitoring value in advanced plasma cell neoplasms.

## Conclusion

The principal lesson of this case is that new-onset hypereosinophilia in relapsed multiple myeloma should not be reflexively attributed to drug toxicity or reactive causes: when eosinophil kinetics track disease burden, eosinophilia may serve as a real-time clinical indicator of aggressive clonal evolution and should trigger prompt evaluation for leukemic transformation and for eosinophil-mediated organ damage, particularly cardiac involvement. By providing what is, to our knowledge, the first detailed CMR characterization of Loeffler-type myocarditis arising in secondary plasma cell leukemia, this report adds a recognizable, non-invasive imaging phenotype to a literature that has so far consisted of isolated reports without cardiac correlates. For clinicians, the actionable message is to integrate eosinophil monitoring and early cardiac imaging into the assessment of biologically aggressive plasma cell disease, since timely cytoreductive therapy may avert irreversible myocardial injury even when the overall prognosis of secondary plasma cell leukemia remains poor.

## Data Availability

No datasets were generated or analysed during the current study.
